# Bibliometric review of worldwide scientific literature on mother-to-child transmission of HIV (1980–2023)

**DOI:** 10.1097/MD.0000000000049182

**Published:** 2026-06-05

**Authors:** Reema A. Karasneh, Sayer I. Al-Azzam, Karem H. Alzoubi, Saed H. Zyoud, Waleed M. Sweileh

**Affiliations:** a Department of Basic Medical Sciences, Faculty of Medicine, Yarmouk University, Irbid, Jordan; b Department of Clinical Pharmacy, Faculty of Pharmacy, Jordan University of Science and Technology, Irbid, Jordan; c Department of Pharmacy Practice and Pharmacotherapeutics, College of Pharmacy, The University of Sharjah, Sharjah, United Arab Emirates; d Faculty of Pharmacy, Jordan University of Science and Technology, Irbid, Jordan; e Department of Physiology and Pharmacology/Toxicology, Division of Biomedical Sciences, College of Medicine and Health Sciences, An-Najah National University, Nablus, Palestine.

**Keywords:** Africa, HIV, research activity, mother-to-child transmission

## Abstract

**Background:**

Transmission of the human immunodeficiency virus (HIV) from HIV-positive mothers to their children (vertical transmission) is a global and serious health problem. Assessing research activity on mother-to-child transmission (MTCT) of HIV helps identify the impact of preventive policies implemented to achieve international goals. The objective of the current study was to assess and analyze research trends and patterns of scientific literature on the MTCT of HIV.

**Methods:**

A bibliometric methodology was applied to literature retrieved from the Scopus database using a pre-validated search strategy. The study period was from 1980 to 2023. Descriptive bibliometric indicators and visualization maps were presented.

**Results:**

The search query found 4468 documents with an *h*-index of 133. Growth of publications based on geographic region showed that publications from the African region have surpassed those from the European Union but are yet to catch up with publications from the Americas. Multiple African countries were also in the top 10 active list of countries, including South Africa (14.8%), Kenya (5.5%), Uganda (4.7%), Nigeria (4%), and Malawi (3.7%). Antiretroviral therapy, newborn and child HIV transmission, and health services were the main research themes encountered. These covered zidovudine therapy, breastfeeding, psychology, and counseling. Zidovudine was also a focus of the top 10 cited documents. PLOS One (n = 216; 4.8%) was the most active journal, while the US CDC (n = 301; 6.7%) was the most active institution in this field.

**Conclusion:**

Ending the MTCT of HIV requires continuous research and collaboration with world regions with high HIV epidemics. High-burden countries, particularly in African and Southeast Asian regions, need to lead the research activity in this field. The recent decline in the number of people with HIV should not decrease research momentum in this field. Challenges remain in increasing HIV treatment coverage, preventing transmission during breastfeeding, and treating infected children.

## 1. Introduction

Acquired human immunodeficiency syndrome (AIDS) and its causative agent, the human immunodeficiency virus (HIV), were medically recognized in the early 1980s.^[1]^ HIV can be transmitted in several ways. Unprotected sexual behaviors are responsible for the majority of new HIV infections. Mother-to-child transmission (MTCT) of HIV accounts for approximately 10%, while the remaining cases are due to transfusion of contaminated blood or the use of contaminated devices.^[2]^ Despite the decline in the global incidence of HIV over the past decade, the high risk of HIV infection remains a challenge among girls and young women, especially in sub-Saharan Africa. According to Joint United Nations Programme on HIV/AIDS (UNAIDS), there were 20.2 million girls and women infected with HIV in 2022, making up more than half of the total HIV cases worldwide.^[3,4]^ MTCT of HIV is responsible for approximately 90% of the new cases of HIV in children, and most of these infected children live in sub-Saharan Africa.^[5]^ MTCT of HIV occurs during pregnancy, labor, delivery, or breastfeeding. Transmission during pregnancy and delivery occurs in 15% to 30% of infants born to untreated, HIV-infected women, whereas 5% to 15% of infants will acquire it via breastfeeding.^[6]^ Other estimates attribute as high as 50% of new pediatric HIV cases to breastfeeding.^[7]^ Transmission of the virus can be significantly reduced upon the use of appropriate antiretroviral therapy (ART).^[8]^ In 2022, failure to receive or adhere to ART was the main cause of vertical HIV transmission, contributing to 94,000 new cases globally. This highlights a looming gap in providing services and treatment coverage for the elimination of MTCT of HIV.^[4]^

Bibliometric analysis is a growing field of information science that has been applied to assess the quantity and trends of research on various medical topics, including HIV/AIDS.^[9]^ In bibliometric analyses, literature from a well-known and large database such as Scopus is retrieved and analyzed concerning evolution, research trends, research gaps, international research collaboration, key players in the field, and important themes being discussed.^[10]^ Bibliometric studies are carried out based on the assumption that the analysis findings can be an important source of information and can lead to the advancement of science. Several bibliometric studies have discussed different aspects of HIV/AIDS. Fajardo-Ortiz et al carried out an analysis of research on HIV/AIDS. They concluded that the most prominent areas of HIV literature were related to molecular mechanisms, drug development, and disease cytopathology.^[11]^ A second bibliometric study on AIDS/HIV has shown a change in research hotspots from prevention of occupational to nonoccupational HIV exposure over the years.^[12]^ A third study investigated HIV stigma and indicated that research on HIV-related stigma has undergone recent growth, with about 40% of documents being published between 2013 and 2017.^[9]^ A fourth study indicated that most research on HIV/AIDS in Africa was dedicated to estimating disease burden, while the remaining focused on prevention and national disease management plans.^[13]^ More recently, Gatasi et al analyzed the top 100 most cited papers on HIV published from 2010 to 2020. US-based universities and health centers dominated the top 10 cited institutions, and transmission and ART were prominent themes.^[14]^

Other HIV/AIDS research areas analyzed using bibliometric methods included medication adherence, health behavioral theories, depression among HIV/AIDS patients, exercise, and classes of drug therapy, such as capsid inhibitors.^[15–18]^ To date, none of the published analyses have been carried out to assess the literature on MTCT of HIV. Therefore, the main objective of the current study was to assess and analyze the literature published on MTCT of HIV. The findings of this study will shed light on the evolution, volume, and scope of research on MTCT and help identify countries and regions lagging in this field. Research output in a certain subject can also be used as an indicator of global and regional efforts to minimize the public health threat of HIV/AIDS.

## 2. Methods

A bibliometric approach was applied. SciVerse Scopus was used to carry out the current study since it is a comprehensive and large database compared with other databases, such as PubMed or Web of Science.^[19]^ The study period was defined as being from 1980 to 2023.

The search strategy utilized the following keywords along with HIV-related keywords: “mother-to-child” or intrapartum or postnatal or antenatal or “mother*” or “breast feed*” or “childbirth” or “lactating mother*” or “lactating women” or “vertical HIV” or “Perinatal HIV*” or “breast feeding” or maternalor perinatal or pregnant or pregnancy or pediatric or infant or newborn or neonate or child or baby or “antiretrovi* therap*”. These keywords were identified based on an extensive literature review of relevant articles.^[20]^ The full search string is as follows: TITLE-ABS (“HIV” OR “human immunodeficiency virus”) AND TITLE-ABS (transmission OR vertical OR “maternal to child*” OR “mother to child” OR maternal)AND TITLE-ABS (pregnan* OR treatment OR prevention OR mother OR maternal OR child OR perinatal) AND (((((((((TITLE (“mother-to-child” OR intrapartum OR postnatal OR antenatal OR “breast feed*” OR “childbirth” OR “lactating mother*” OR “lactating women” OR “vertical HIV” OR “Perinatal HIV*” OR “breast feeding” OR (maternal AND infant) OR (maternal AND “viral load”) OR “perinatal exposure” OR “perinatal transmission”) AND TITLE (hiv-* OR hiv* OR aids OR “antiretrovi* therap*” OR hiv-1 OR “Acquired Immunodeficiency Syndrome” OR “human immunodeficiency virus” OR zidovudine OR nevirapine OR “protease inhibit*” OR antiretroviral OR lamivudine OR tenofovir OR ritonavir OR efavirenz OR stavudine OR zidovudine) AND TITLE-ABS (virus OR hiv OR immuno*) AND TITLE-ABS (“mother to child*” OR transmission OR “maternal-infant” OR vertical))) OR (TITLE (pregnant OR pregnancy OR child* OR newborn OR baby OR neonatal OR pediatric OR infant) AND TITLE (hiv-* OR hiv* OR aids OR antiretrovir* OR hiv-1 OR “Acquired Immunodeficiency Syndrome”) AND TITLE-ABS (“mother-to-child*” OR “lactating mother*” OR “lactating women” OR breastfeeding OR “maternal-infant”) AND TITLE-ABS (transmission OR vertical) AND ALL (mother* OR maternal))) AND NOT TITLE (haemophil*) AND NOT TITLE (hepatitis))))))) AND PUBYEAR>1980 AND PUBYEAR<2024 AND (LIMIT-TO (SRCTYPE, “j”)) AND (EXCLUDE (DOCTYPE, “er”) OR EXCLUDE (DOCTYPE, “le”) OR EXCLUDE (DOCTYPE, “no”) OR EXCLUDE (DOCTYPE, “cp”) OR EXCLUDE (DOCTYPE, “ed”)).

### 2.1. Inclusion and exclusion criteria

**Figure 1.**
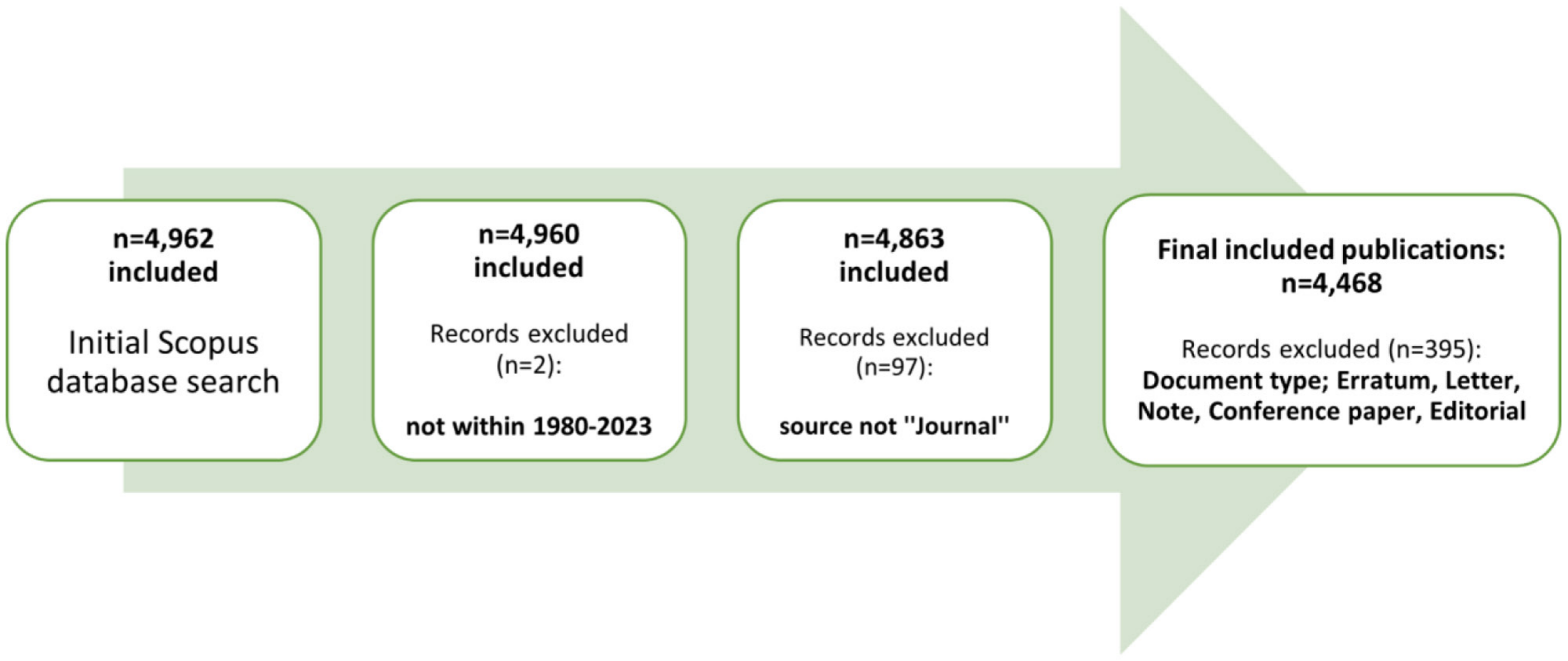
Screening process for documents on MTCT of HIV to be included in the study. HIV = human immunodeficiency virus, MTCT = mother-to-child transmission.

Only documents published in “journals” were included. Documents were excluded if they were published outside of the study period (1980–2023). The following document types were excluded: Erratum, Letter, Note, Conference paper, and Editorial (Fig. 1).

The search strategy was validated for the absence of false-positive documents by scanning the titles of the top 50 cited documents in the retrieved literature. A false-positive rate of <5% was accepted. Microsoft Excel (Microsoft Corporation) was used to create graphs, while VOSviewer software (Leiden University)^[21]^ was used to visualize relevant results. The degree of author collaboration was calculated using the following formula: Degree of collaboration = C = Nm/Nm + Ns, where Nm = number of multi-authored papers and Ns = number of single-authored papers.^[22,23]^ To ensure standardization, we utilized a recently developed guideline for reporting bibliometric reviews, BIBLIO.^[24]^ The guideline outlines minimum requirements for accurate reports of bibliometric analyses, including a 20-item checklist.

Bibliometric indicators used in the current study included the following:

1. General characteristics of the retrieved literature, including citation analysis and H-index,^[25]^ which measures the impact of a certain document or subject.
2. Annual growth and distribution of publications based on World Health Organization (WHO) regions: the region of the Americas, the European region, the African region, the Eastern Mediterranean region, the Southeast Asian region, and the Western Pacific region.
3. Visualizations of frequent terms in the title/abstract to spot the presence of research themes and to find out the most encountered medications.
4. Top 10 active countries.
5. Top 10 active journals.
6. Top 10 active institutions/organizations.
7. Visualization of active authors with a minimum of 30 publications.
8. Top 10 cited documents were presented.

## 3. Results

### 3.1. General characteristics of the retrieved literature

The search was conducted in January 2024 and found 4468 documents. Research articles (85.4%; n = 3816) constituted the majority of the retrieved documents. Other retrieved documents included review articles (13.7%; n = 614) and short surveys (0.9%; n = 38). The retrieved documents were cited 118,350 times, averaging 26.5 citations per document. The *h*-index of the retrieved documents was 133.

### 3.2. Annual growth of publications and distribution based on WHO regions

**Figure 2.**
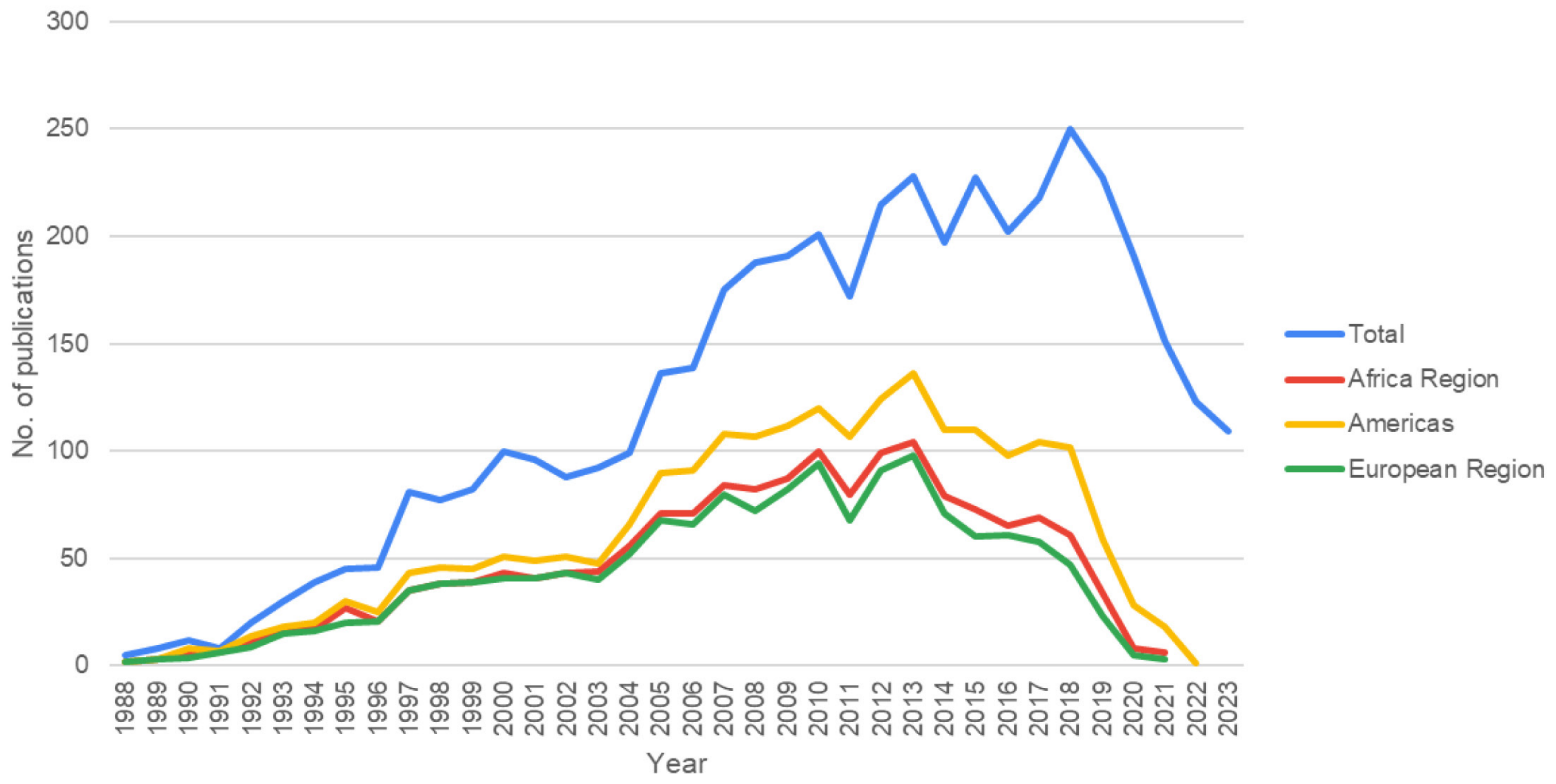
Annual growth of publication on MTCT of HIV 1980–2023. HIV = human immunodeficiency virus, MTCT = mother-to-child transmission.

The retrieved documents showed a fluctuating upward increase from the early 1990s until 2000, followed by a period (2000–2004) of a constant number of publications, approximately 100 documents per year (Fig. 2). The oldest documents related to MTCT of HIV appeared in 1988. Analysis of the retrieved literature based on WHO geographic region indicated that authors from the African region participated in publishing 2593 (58%) documents, the region of the Americas participated in 2365 (52.9%), the European region participated in 1824 (40.8%), the Western Pacific region participated in 299 (6.7%), the South-Eastern Asian region participated in 290 (6.5%), and the Eastern Mediterranean region participated in 30 (0.7%) documents. Figure 2 shows the growth of publications from the African region exceeding that of the European region from 2005 to 2020. However, they remain lagging behind the Americas region. A wide gap in African publications was found when limiting search results to African region countries only (−971 documents), suggesting more than half of the publications constitute collaborations with other regions.

### 3.3. Authorship analysis

**Figure 3.**
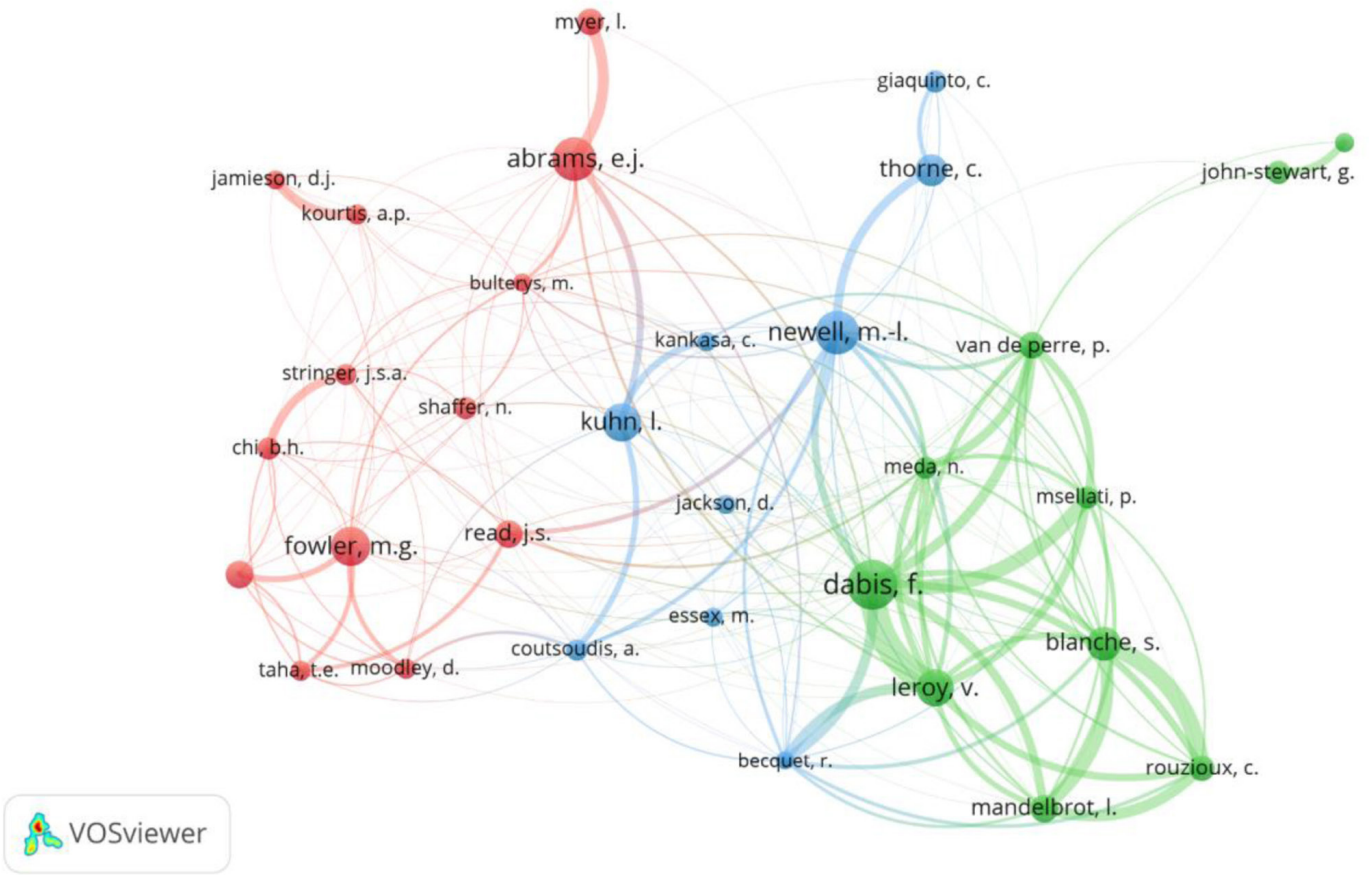
Author collaborations among active authors with a minimum of 30 publications.

In total, 16,446 authors participated in publishing the retrieved documents, giving an average of 3.7 authors per document. Of the retrieved documents, 265 (5.9%) were single-authored publications. The degree of collaboration was 94%. Dabis, F was the most prolific author (n = 97, 2.2%), followed by Newell, ML (n = 83, 1.9%) and Fowler, MG (n = 74, 1.7%). All 3 authors collaborated, as illustrated in Figure 3.

### 3.4. Research themes and commonly encountered medications

**Figure 4.**
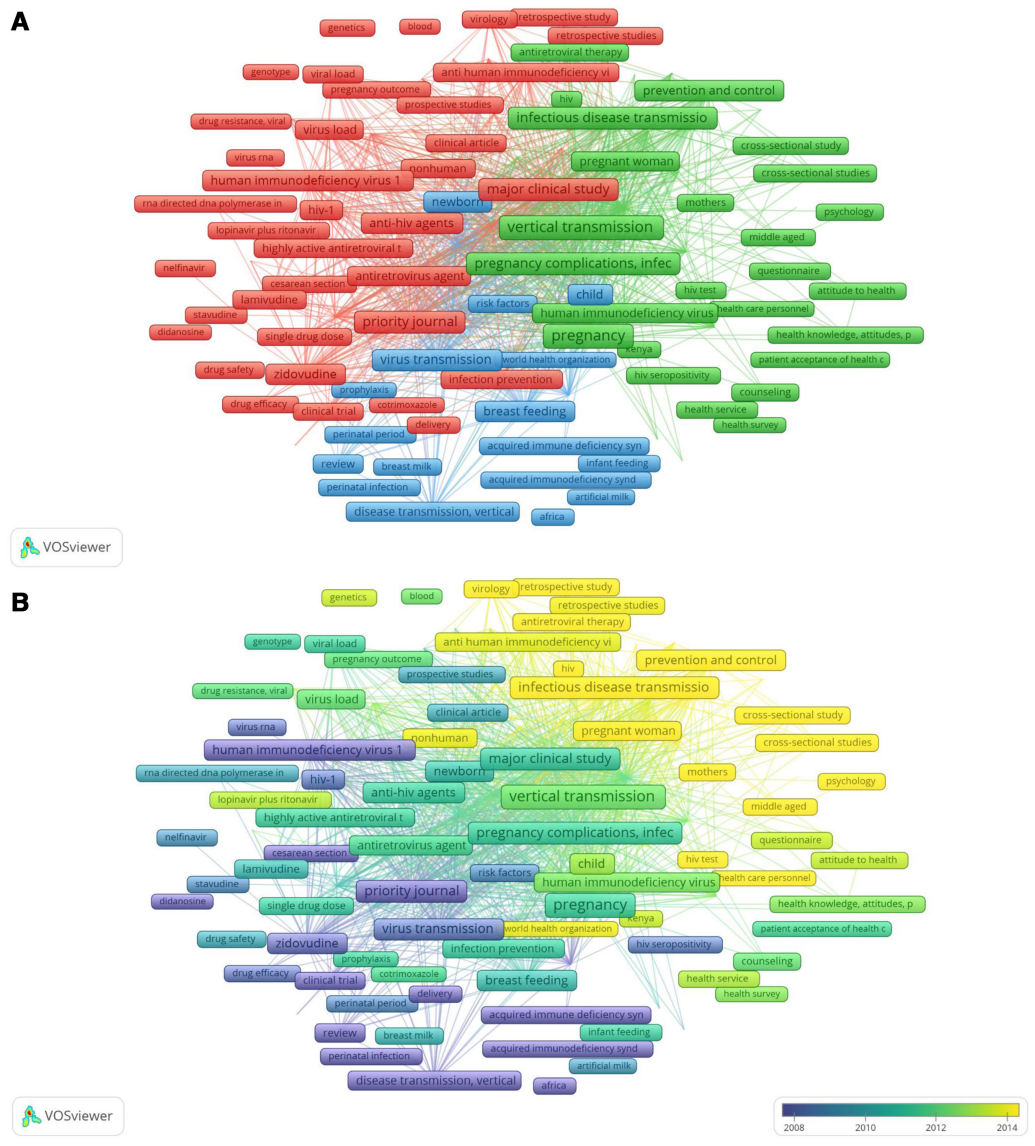
Thematic and temporal analysis of the retrieved publications. (A) Research themes based on terms commonly encountered in titles/abstracts of the retrieved literature. The map was created by VOSviewer and includes the top 150 frequent keywords. Each cluster of terms represents a research theme. (B) An overlay visualization of research themes based on terms commonly encountered in titles/abstracts of the retrieved literature. The map was created by VOSviewer and includes the top 150 frequent keywords. Each cluster of terms represents a research theme. The overlay visualization reflects the average publication year of keywords rather than the full search period; therefore, the displayed range (2008–2014) corresponds to the years in which the majority of indexed terms in the network are concentrated.

Visualization of the 150 most frequent terms in titles/abstracts of the retrieved literature indicated the presence of 3 major research themes in the field: the red and largest cluster represented the research theme on antiretroviral therapies (Fig. 4A). Analysis of indexed keywords showed that zidovudine (ZDV; n = 1,195) was the most commonly mentioned drug, followed by lamivudine (n = 568), efavirenz (n = 238), lopinavir plus ritonavir (n = 214), ritonavir (n = 202), stavudine (n = 190), nelfinavir (n = 188), tenofovir (n = 176), didanosine (n = 166), co-trimoxazole (n = 163), and nevirapine (n = 104). The overlay map (Fig. 4B) shows publications on ZDV and didanosine in a darker color, dating back to before 2010, whereas publications on lopinavir plus ritonavir were the most recent. RNA-directed DNA polymerase inhibitors were the most commonly encountered as a drug class, followed by protease inhibitors.

The green cluster shows a theme related to health services and care offered for HIV-positive mothers/pregnant women. Research topics within this cluster, such as counseling, psychology, prevention, and control, were relatively more recent than research on antiretroviral regimens. Meanwhile, the blue and smallest cluster focused on newborn children of HIV-positive mothers and breastfeeding.

### 3.5. Active countries and research collaboration

Authors from 131 countries on 6 continents contributed to the retrieved documents. The top 10 active countries are shown in Table 1. The top 10 countries contributed to approximately 99.4% (n = 4443) of the retrieved documents. The USA (41.9%; n = 1874) led in terms of the number of publications, followed by South Africa (14.8%; n = 661) and the United Kingdom (10.2%; n = 457). The top 10 list included 5 countries in Africa, 3 in Europe, and 2 in North America.

**Table 1:** Top 10 active countries and extent of international collaboration on MTCT of HIV.

Rank	Country	Number of publications (N = 4468)	%	%
1st	United States	1874	41.9	42.5
2nd	South Africa	661	14.8	56.8
3rd	United Kingdom	457	10.2	41.6
4th	France	325	7.3	29.9
5th	Kenya	246	5.5	60.1
6th	Uganda	210	4.7	25.9
7th	Nigeria	178	4	50.7
8th	Canada	166	3.7	52.5
9th	Malawi	164	3.7	54.4
10th	Italy	162	3.6	67.1

The first percentage column represents the contribution from retrieved publications, while the second percentage column represents the proportion of publications with international collaboration, for each country.

HIV = human immunodeficiency virus, MTCT = mother-to-child transmission.

### 3.6. Top 10 active journals

The top 10 active journals in publishing documents on MTCT of HIV are listed in Table 2. PLOS One journal led with 216 (4.8%) documents, followed by AIDS with 215 (4.8%) documents. The total number of documents published by the top 10 active journals was 1150 (25.7%). Documents published in the *Journal of Infectious Diseases* and AIDS received the highest number of citations per document: 73.9 and 57.8, respectively.

**Table 2:** Top 10 active journals in publishing documents on MTCT of HIV.

Rank	Journal	Number of publications (N = 4468)	%	Total citations	Citation per document
1st	PLOS One	216	4.8	4134	19.1
2nd	AIDS	215	4.8	12,422	57.8
3rd	Journal of Acquired Immune Deficiency Syndromes	212	4.7	9074	42.8
4th	BMC Public Health	89	2	1771	19.9
5th	AIDS Care Psychological and Socio Medical Aspects of AIDS HIV	83	1.9	1598	19.3
6th	Journal of Infectious Diseases	79	1.8	5841	73.9
7th	Journal of The International AIDS Society	75	1.7	2412	32.2
8th	Pediatric Infectious Disease Journal	64	1.4	1175	18.4
9th	AIDS Research and Human Retroviruses	63	1.4	931	14.8
10th	International Journal of STD and AIDS	54	1.2	543	10.1

HIV = human immunodeficiency virus, MTCT = mother-to-child transmission.

### 3.7. Top 10 active institutions/organizations

Table 3 shows the top 10 active institutions. The list included 2 nonacademic institutions and 8 academic institutions. Institutions from the USA dominated the list, and the *Centers for Disease Control and Prevention (CDC*), a research institute in the USA, was the top active institution (6.7%; n = 301). The top 10 list included 3 South African institutions and 1 from France. The remaining institutions were based in the USA.

**Table 3:** Top 10 active institutions in publishing documents on MTCT of HIV.

Rank	Institutions	Number of publications (N = 4468)	%	Country
1st	Centers for Disease Control and Prevention	301	6.7	USA
2nd	University of Witwatersrand	197	4.4	South Africa
3rd	Inserm	174	3.9	France
4th	Columbia University	172	3.8	USA
5th	Harvard T.H. Chan School of Public Health	159	3.6	USA
6th	University of Washington	155	3.5	USA
7th	University of KwaZulu-Natal	147	3.3	South Africa
8th	University of Cape Town	140	3.1	South Africa
9th	The University of North Carolina at Chapel Hill	138	3.1	USA
10th	Johns Hopkins University	131	2.9	USA

HIV = human immunodeficiency virus, MTCT = mother-to-child transmission.

### 3.8. Top 10 cited documents

Table 4 shows the top 10 cited documents. Five were published in *The Lancet,* and 4 discussed the role of ZDV in preventing vertical transmission and its link to toxic effects; 1 compared nevirapine to ZDV, and 1 discussed the role of combination therapy.^[26–35]^ All top 10 cited documents were published before 2010. For documents published after 2005, the most cited documents focused on similar issues, such as therapy with ZDV, nevirapine, and combination therapy.

**Table 4:** Top 10 cited documents on MTCT of HIV.

Title	Year	Source title	Number of citations
Reduction of maternal-infant transmission of human immunodeficiency virus type 1 with zidovudine treatment	1994	*New England Journal of Medicine*	3389
Intrapartum and neonatal single-dose nevirapine compared with zidovudine for prevention of mother-to-child transmission of HIV-1 in Kampala, Uganda: HIVNET 012 randomised trial	1999	*Lancet*	1480
Mortality of infected and uninfected infants born to HIV-infected mothers in Africa: a pooled analysis	2004	*Lancet*	932
Combination antiretroviral strategies for the treatment of pregnant HIV-1-infected women and prevention of perinatal HIV-1 transmission	2002	*Journal of Acquired Immune Deficiency Syndromes*	872
Prevention of mother-to-child HIV transmission in resource-poor countries: Translating research into policy and practice	2000	*Journal of the American Medical Association*	813
Maternal viral load, zidovudine treatment, and the risk of transmission of human immunodeficiency virus type 1 from mother to infant	1996	*New England Journal of Medicine*	704
Short-course zidovudine for perinatal HIV-1 transmission in Bangkok, Thailand: a randomised controlled trial	1999	*Lancet*	679
Maternal levels of plasma human immunodeficiency virus type 1 RNA and the risk of perinatal transmission	1999	*New England Journal of Medicine*	639
Persistent mitochondrial dysfunction and perinatal exposure to antiretroviral nucleoside analogues	1999	*Lancet*	594
Elective caesarean-section versus vaginal delivery in prevention of vertical HIV-1 transmission: a randomised clinical trial	1999	*Lancet*	554

HIV = human immunodeficiency virus, MTCT = mother-to-child transmission.

## 4. Discussion

The current study aimed to assess the research activity on MTCT of HIV, which is 1 component of the fight against HIV. The current study showed that the growth of publications increased linearly and peaked in 2018, falling slightly afterward. UNAIDS has noted that the number of people living with HIV globally is declining in most parts of the world. A significant decline was particularly recorded in eastern and southern Africa and western and central Africa. HIV incidence in these regions saw a reduction in 2022 of 57% and 49%, respectively, compared with 2010.^[4]^

Since 2010, new HIV infections among children have declined by 62%, from 310,000 (220,000–450,000) in 2010 to 120,000 (82,000–170,000) in 2024. Although progress in reducing new HIV infections is greatest among children, progress has stalled in recent years.^[5]^ Furthermore, around 76% of pregnant women living with HIV had access to ART to prevent transmission to their children in 2016. The decline in numbers is the outcome of the tremendous efforts of governments and international health organizations in implementing preventive policies, the increased access to ART in low-resource countries, and the research and advancement of new ART medications.^[5]^ The decline in numbers should not decrease the momentum of the fight against HIV in general. A review of global health challenges published by the WHO in 2019 showed that HIV is still considered a threat to global health.^[36]^ The current study showed that the retrieved documents had an *h*-index above 130, suggesting that this topic interests many researchers and has been cited frequently. The *h*-index of the retrieved documents was slightly lower than the literature on AIDS medication adherence^[15]^ but higher than that on AIDS-related stigma.^[9]^

The number of publications retrieved in the current study can be considered high relative to the volume of literature on vertically transmitted infectious diseases such as herpes simplex virus or rubella (data not shown). The serious nature of HIV disease, the international funding and support, and the struggle to implement and achieve millennium and sustainable development goals helped increase research activity and the number of publications on MTCT of HIV.^[37]^ The current study showed that publications on MTCT of HIV started in the late 1980s, a few years after the spread of the disease in the early 1980s. This gap between the beginning of the disease and research activity has been observed in other topics. For example, the growth of publications on medication adherence to anti-HIV medications started a decade after the start of the general HIV/AIDS literature.^[15]^ In the current study, the retrieved literature mainly addressed therapeutic regimens, prevention, and outcomes in newborns, transmission through breastfeeding, as well as health services and care for HIV-positive mothers.

Since the discovery of the first cases of possible MTCT of HIV, researchers have focused on developing methods to minimize HIV transfer from mother to child. Most of the research on this front focused on how to reduce the maternal viral load and subsequently minimize the transfer of the virus. Results of several studies and clinical trials using ZDV, nevirapine, and cesarean section were translated into guidelines by WHO.^[38]^ The guideline released by WHO in 2016 adopted “Option B+,” in which all pregnant and breastfeeding women living with HIV are recommended to take lifelong ART regardless of their cluster of differentiation 4 count.^[39,40]^

A relatively recent research theme in this analysis is HIV infection in newborns and children born to HIV-infected mothers. The 2023 UNAIDS report identified gaps in the treatment of children living with HIV, including lower rates of ART coverage compared with adults (57% vs 77%) and the need for early infant diagnosis.^[4]^ Early diagnosis and treatment of infected children is a focus of MTCT research, with the risk of mortality estimated at 35% in the first year of life and 50% in the second year for untreated, infected children.^[41]^ Improving and monitoring adherence was also an active topic, prompted by a search for safe, efficacious, and palatable therapies.^[42]^ The LOLIPOP study assessed the acceptability of a 4-in-1 regimen consisting of a combination of granules of abacavir, lamivudine, and lopinavir/ritonavir in children.^[43]^ Results were analyzed in comparison with lopinavir/ritonavir formulations known for a bitter taste affecting adherence. The regimen was found to be more favorable to caregivers regarding palatability and ease of administration. Another single-arm trial in collaboration between South Africa, Uganda, the United States, and Thailand assessed a single-dose ART regimen among children aged 6 to 18.^[44]^ The formulation consisted of bictegravir/emtricitabine/tenofovir alafenamide (50/200/25 mg). The overwhelming majority of participants reported the regimen to be acceptable and palatable.

MTCT via feeding was also a prominent research area in the last 2 years. This momentum in research has helped inform WHO guidelines, which currently recommend that mothers on ART breastfeed for the first 12 months, with the possibility of continuing until or beyond 24 months.^[45]^ Screening for HIV, breastfeeding counseling on best practices, monitoring for ART adherence and HIV viral load, and infant prophylaxis are necessary steps for the elimination of vertical MTCT during breastfeeding. A study from Ethiopia reported high rates of unsafe breastfeeding practices among HIV-positive mothers.^[46]^ Recommendations to decrease the risk of transmission include limiting feeding time, exclusive breastfeeding for 6 months, and managing breast health issues.^[46]^

The current study showed that non-American, non-European countries such as South Africa, Kenya, Uganda, and Malawi (the southern and eastern region of sub-Saharan Africa) made noticeable contributions to the literature on the MTCT of HIV. Recent statistics indicate that more than two-thirds of people living with HIV in the world are living in the African region, which explains the noticeable research output from certain African countries.^[47]^ The second potential explanation for the research output from certain African countries is the struggle to meet the UNAIDS 90-90-90 targets.^[48]^ These efforts reflected positively on the number of new HIV infections, which recorded a 56% decline between 2010 and 2016 among children.^[48]^ The third potential reason for the research contribution by certain African countries is the significant international research collaboration between developing countries and countries in Africa.

### 4.1. Limitations

The current study has a few limitations. First, world regions with a limited number of journals indexed in Scopus will be underrepresented. This is true for the African region, Southeast Asian region, Latin America, and the Eastern Mediterranean region. Scopus is biased toward English journals published in Europe and North America. Therefore, local and regional non-English journals were mostly missed and not included in the analysis. A second limitation is the list of active authors and institutions, which needs to be carefully interpreted due to the overlap in publications, research networking, self-citations, and the mobility of researchers from 1 institution to another. Furthermore, the bibliometric review guideline (BIBLIO) is a preliminary guide that requires further validation for quality and utility.

## 5. Conclusion

In fighting HIV and AIDS, the research area of MTCT of HIV is crucial. If research and preventive policies in this area are ignored, the number of new HIV infections will increase. The current study’s findings will benefit several national and international agencies in prioritizing research funding. Furthermore, the findings of the current study emphasize that high-burden, middle-income countries in Africa and elsewhere must take the lead in the global HIV research agenda through the creation of domestic financial support and international research collaboration. The research output obtained in this study reflects the extent of national knowledge and willingness to implement national preventive measures to fulfill global ambitions in the fight against HIV. Finally, the noticeable success achieved in the past few years in the declining number of people living with HIV should not create a misleading belief that the HIV epidemic is over. The road to eradicating HIV is still long, and research on prevention among mothers and children remains vital.

## Author contributions

**Conceptualization:** Reema A. Karasneh, Sayer I. Al-Azzam, Karem H. Alzoubi, Saed H. Zyoud, Waleed M. Sweileh.

**Data curation:** Reema A. Karasneh, Sayer I. Al-Azzam, Karem H. Alzoubi, Saed H. Zyoud, Waleed M. Sweileh.

**Formal analysis:** Reema A. Karasneh.

**Investigation:** Reema A. Karasneh, Sayer I. Al-Azzam, Karem H. Alzoubi, Saed H. Zyoud, Waleed M. Sweileh.

**Methodology:** Reema A. Karasneh, Saed H. Zyoud.

**Funding acquisition:** Sayer I. Al-Azzam.

**Validation:** Sayer I. Al-Azzam, Waleed M. Sweileh.

**Project administration:** Reema A. Karasneh, Sayer I. Al-Azzam, Karem H. Alzoubi, Saed H. Zyoud, Waleed M. Sweileh.

**Supervision:** Reema A. Karasneh, Karem H. Alzoubi, Waleed M. Sweileh.

**Visualization:** Reema A. Karasneh, Saed H. Zyoud, Waleed M. Sweileh.

**Writing – original draft:** Reema A. Karasneh, Sayer I. Al-Azzam, Karem H. Alzoubi, Saed H. Zyoud, Waleed M. Sweileh.

**Writing – review & editing:** Reema A. Karasneh, Sayer I. Al-Azzam, Karem H. Alzoubi, Waleed M. Sweileh.
